# Removal of beneficial insertion effects prevent the long-term persistence of transposable elements within simulated asexual populations

**DOI:** 10.1186/s12864-021-07569-3

**Published:** 2021-04-07

**Authors:** Christopher L. Butler, Ellen A. Bell, Martin I. Taylor

**Affiliations:** School of Biological Sciences, University of East Anglia, Norwich Research Park, Norwich, NR4 7TJ UK

**Keywords:** Transposon proliferation, Genome ecology, Positive selection, C-value, In-silico model

## Abstract

**Background:**

Transposable elements are significant components of most organism’s genomes, yet the reasons why their abundances vary significantly among species is poorly understood. A recent study has suggested that even in the absence of traditional molecular evolutionary explanations, transposon proliferation may occur through a process known as ‘transposon engineering’. However, their model used a fixed beneficial transposon insertion frequency of 20%, which we believe to be unrealistically high.

**Results:**

Reducing this beneficial insertion frequency, while keeping all other parameters identical, prevented transposon proliferation.

**Conclusions:**

We conclude that the author’s original findings are better explained through the action of positive selection rather than ‘transposon engineering’, with beneficial insertion effects remaining important during transposon proliferation events.

## Background

Transposable elements (TEs) are short regions of non-coding DNA (100-10,000 bp) which can proliferate throughout a genome, and are significant genomic components of a taxonomically diverse range of species [1–3]. Understanding the processes which drive TE variation across different species is an important goal in answering the so-called ‘C-value’ paradox (the observed lack of relationship between genome size and organismal complexity) [4]. TEs are thought to accumulate within a population through a number of evolutionary mechanisms which include (i) positive selection, (ii) genetic drift, (iii) co-evolution with the host, (iv) sexual recombination or (v) horizontal transfer. Kremer et al. [5] investigated the fate of TE populations where none of these population genetic scenarios were possible [5]. Using in-silico modelling, the authors established an asexual population where (i) TE insertions had serious negative effects on host fitness, (ii) TEs could not evolve insertion site preferences (i.e. no co-evolution with the host) and (iii) TEs were not able to be horizontally transferred. Surprisingly, even in the absence of these evolutionary forces, TEs accumulated in a limited number (3%) of scenarios. The authors concluded that these rare accumulation events may be explained through ‘TE engineering’; a process in which the activity of TEs significantly alters the landscape of a genome to facilitate further proliferation. Specifically, they suggest that the cycle of TE proliferation and degradation may provide new non-coding regions in which future TEs can insert with little or no consequence on host fitness. Changes in TE abundance which occur through their interactions with either the host genome or other transposons comprise a poorly studied field known as ‘TE ecology’ [6, 7]. Consequently, Kremer et al. [5] appears to have identified a novel mechanism for TE proliferation, with important implications regarding our understanding of TE dynamics.

Here, we highlight some potential issues which question the key findings of Kremer et al’s study. They claim that their simulations model TE insertions which do not have any beneficial effects on host fitness; crucially ruling out positive selection as an explanation for TE accumulation. However, there was a lack of clarity on the precise meaning of ‘no beneficial effects’, with three explanations on the effects of TE insertions given in their paper. These were: (i) ‘TEs had a net deleterious effect on host fitness’, (ii) TEs had ‘serious negative effects on host fitness’ or, (iii) ‘violated the assumption that TE insertions are beneficial’. A model in which TE activity had a net deleterious effect could mean that only a very small majority of TE insertions reduce host fitness. Such a model would violate the author’s own assumption that TE insertions cannot be beneficial, with fitness increases still occurring during a significant number of insertions. Greater clarity on this issue would have been beneficial in order to help validate the legitimacy of Kremer et al’s conclusions.

The final model used in Kremer et al. [5] included a fixed parameter which simulates a mildly beneficial fitness effect (Insertion_effect) during 20% of all TE insertions. Crucially, setting an insertion benefit at this level is not consistent with the author’s own conclusion that positive selection is not responsible for driving the TE accumulation events observed. Theoretically, the level of beneficial TE insertions may not have to be very high for their gradual accumulation. The fact that original TE copies are frequently retained in the genome (i.e. progeny distribution >1) can provide a buffer to their abundance, even if the majority of new insertions are deleterious. This has been demonstrated in other simple TE dynamic models, whereby increasing the adaptive insertion probability to 0.05% is sufficient to permit TE domestication through positive selection [8]. In this study, we repeated Kremer et al’s [5] simulations, but explicitly defined ’no beneficial insertion effect’ to mean there was no scenario in which TE insertions could generate an increase in host fitness, thus definitely ruling out positive selection as a potential mechanism for TE proliferation.

## Methods

To test whether removing the positive TE insertion effect altered their ability to accumulate, we reanalysed the six parameter scenarios from Kremer et al. [5] where in the majority of cases TEs persisted for the 1,500 generation cut off set by the authors (Table 1). We did not change the percentage of TE insertions which led to lethal deleterious (20%) or mildly deleterious (30%) fitness effects. Instead we increased the probability that a TE insertion was neutral (i.e generating no change in host fitness) from 30% to 50%. All other model parameters remained identical. Following Kremer et al., we then repeated each simulation three times independently, which were plotted using the ‘ggplot2’ package in R v3.5.1 [9].

**Table 1:** The parameter scenarios where Kremer et al. [5] reported TE accumulation in the majority of cases

TE Properties				Host Properties				Parameter Scenario
TE Progeny	TE Excision Rate	TE Death Rate	Insertion Bias	Corrected Mutation Rate	Non-Coding DNA	Mutation Effect	Carrying Capacity	
High	Low	Low	Low	Low	High	Low	High	HLLL - LHLH
Low	Low	High	High	Low	High	High	High	LLHH - LHHH
Low	Low	Low	High	Low	High	High	Low	LLLH - LHHL
Low	Low	Low	High	Low	Low	High	High	LLLH - LLHH
Low	Low	Low	Low	Low	High	High	High	LLLL - LHHH
Low	Low	Low	Low	Low	Low	High	Low	LLLL - LLHL

## Results

**Fig. 1:**
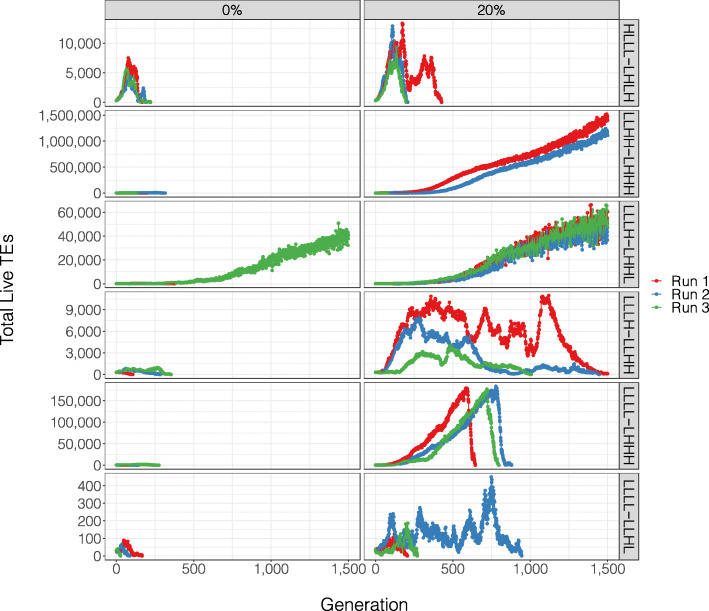
TE population dynamics for each of the six parameters where Kremer et al. [5] reported TE accumulation in the majority of cases. Beneficial TE insertions were set at either 0% or 20%. When beneficial insertion effects were excluded from the model, TEs failed to proliferate in all but one case (Run 3 of LLLH-LHHL)

**Fig. 2:**
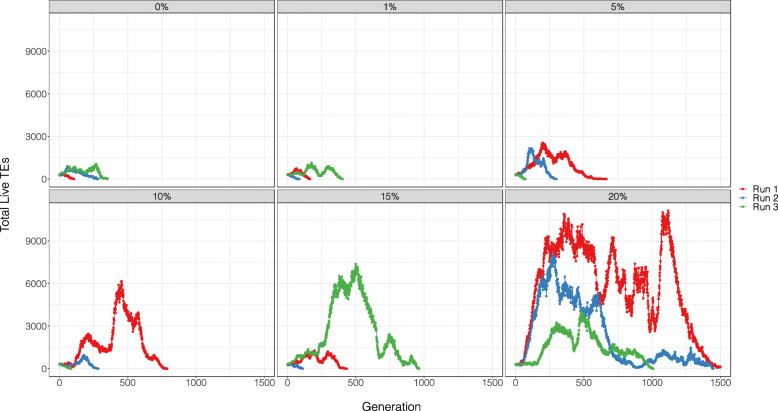
TE population dynamics when the beneficial insertion frequency were set at 0%, 1%, 5%, 10%, 15% and 20% respectively. TEs were only able to accumulate for 1,500 generations when the positive insertion frequency was set at 20%. In all other scenarios TE extinction occurred before the simulation ran to completion. The parameter setting for this iteration was LLLH-LLHH

In our reanalysis, we found that across each of the six parameter scenarios, TEs were better able to accumulate when the model included a beneficial TE insertion effect (Fig. 1). Indeed, in the 18 iterations we ran, TEs only accumulated in a single instance when the beneficial insertion effects were removed (Run 3 of LLLH-LHHL). We also investigated the frequency of beneficial TE insertions that would be required for TE accumulation. To do this, we chose a parameter scenario in which Kremer et al. found TEs to accumulate in every run (namely LLLH-LLHH). When the beneficial TE insertion effect was reduced to 0 (from 20%), the TE population did not proliferate, becoming extinct in under 400 generations in every iteration. We also ran simulations where TE insertions increased host fitness 1%, 5%, 10% and 15% of the time. While this led to incremental increases in TE accumulation, none of the simulations lasted 1,500 generations (Fig. 2). We therefore conclude that under this TE dynamic model, a significant positive insertion effect is required for TE accumulation in almost all cases.

## Discussion

Overall, our findings suggest that Kremer et al’s [5] key conclusion of TE accumulation in a significant number of cases can largely be explained by the high beneficial insertion frequency used by the authors. When we removed this effect, TEs did not persist in the overwhelming majority of scenarios. We therefore suggest that the author’s original finding of TE accumulation would have been better explained by the action of positive selection instead of a ‘TE engineering’ process. The 20% beneficial insertion effect set by the authors is likely to be a significant overestimate compared to what may be observed in reality. Whilst estimating the frequency of beneficial insertion effects remains difficult, a recent genome-wide scan of 14,384 human TE polymorphisms concluded that just 1.13% (163) were under positive selection [10]. The true frequency of beneficial TE insertions is likely to be even lower, as many highly deleterious or neutral TEs will lie undetected as they are removed from the genome. Interestingly, we did identify a single combination of parameters in which TEs did accumulate without exhibiting any beneficial fitness effect; namely when TE progeny and excision rate is low, TEs display high insertion bias, and the degree of non-coding regions in the genome are high. This may provide an interesting avenue for understanding genomic characteristics where TEs may accumulate in the absence of positive selection. Many TEs display both an insertion site bias [11, 12] and variable TE activity rates (a product of both TE excision and progeny rates) within the lifetime of a cell [13]. Finally, we wish to emphasise that we are not suggesting that TE ecology explanations should be ruled out when trying to understand the reasons for TE accumulation. On the contrary, when exploring potential hypotheses for TE proliferation it is important to realise that both evolutionary and ecology processes are likely to occur concurrently.

## Data Availability

The datasets analysed during this study were generated using the TE_World2 model, which can be accessed at: https://github.com/stefan-c-kremer/TE_World2 [5]. Declarations

## Abbreviations

TE: Transposable element
